# Protein profiling and assessment of amyloid beta levels in plasma in canine refractory epilepsy

**DOI:** 10.3389/fvets.2023.1258244

**Published:** 2023-12-21

**Authors:** Sataporn Phochantachinda, Boonrat Chantong, Onrapak Reamtong, Duangthip Chatchaisak

**Affiliations:** ^1^Department of Clinical Sciences and Public Health, Faculty of Veterinary Science, Mahidol University, Nakhon Pathom, Thailand; ^2^Department of Pre-Clinic and Applied Animal Science, Faculty of Veterinary Science, Mahidol University, Nakhon Pathom, Thailand; ^3^Department of Molecular Tropical Medicine and Genetics, Faculty of Tropical Medicine, Mahidol University, Bangkok, Thailand

**Keywords:** amyloid beta, epilepsy, proteomics, canine, signaling pathways

## Abstract

**Introduction:**

The relationship between epilepsy and cognitive dysfunction has been investigated in canines, and memory impairment was prevalent in dogs with epilepsy. Additionally, canines with epilepsy have greater amyloid-β (Aβ) accumulation and neuronal degeneration than healthy controls. The present study investigated plasma Aβ_42_ levels and performed proteomic profiling in dogs with refractory epilepsy and healthy dogs.

**Methods:**

In total, eight dogs, including four healthy dogs and four dogs with epilepsy, were included in the study. Blood samples were collected to analyze Aβ_42_ levels and perform proteomic profiling. Changes in the plasma proteomic profiles of dogs were determined by nano liquid chromatography tandem mass spectrometry.

**Results and discussion:**

The plasma Aβ_42_ level was significantly higher in dogs with epilepsy (99 pg/mL) than in healthy dogs (5.9 pg/mL). In total, 155 proteins were identified, and of these, the expression of 40 proteins was altered in epilepsy. Among these proteins, which are linked to neurodegenerative diseases, 10 (25%) were downregulated in dogs with epilepsy, whereas 12 (30%) were upregulated. The expression of the acute phase proteins haptoglobin and α2-macroglobulin significantly differed between the groups. Complement factor H and ceruloplasmin were only detected in epilepsy dogs, suggesting that neuroinflammation plays a role in epileptic seizures. Gelsolin, which is involved in cellular processes and cytoskeletal organization, was only detected in healthy dogs. Gene Ontology annotation revealed that epilepsy can potentially interfere with biological processes, including cellular processes, localization, and responses to stimuli. Seizures compromised key molecular functions, including catalytic activity, molecular function regulation, and binding. Defense/immunity proteins were most significantly modified during the development of epilepsy. In Kyoto Encyclopedia of Genes and Genomes pathway analysis, complement and coagulation cascades were the most relevant signaling pathways affected by seizures. The findings suggested that haptoglobin, ceruloplasmin, α2-macroglobulin, complement factor H, and gelsolin play roles in canine epilepsy and Aβ levels based on proteomic profiling. These proteins could represent diagnostic biomarkers that, after clinical validation, could be used in veterinary practice as well as proteins relevant to disease response pathways. To determine the precise mechanisms underlying these relationships and their implications in canine epilepsy, additional research is required.

## 1 Introduction

Epileptic seizures are described by the International Veterinary Epilepsy Task Force (IVETF) as “a transient occurrence of signs due to abnormal excessive or synchronous neuronal activity in the brain” (1). The prevalence of canine epilepsy has been estimated to be 0.6–0.75% in the general dog population (2). Canine seizures significantly affect the health and well-being of dogs as well as their owners. Repeated or prolonged seizures can cause damage to the brain and nervous system, which can lead to cognitive and behavioral problems, as well as increased seizure frequency and severity (3, 4). Dogs that experience seizures can injury themselves or others during an episode because of the loss of consciousness, loss of motor control, or other factors (3, 4). Seizures can be emotionally stressful for both dogs and their owners, and they can lead to anxiety, depression, and decreased quality of life (5).

The pathophysiology of canine seizures is complex and incompletely understood. However, it is believed to involve a disruption in the balance of excitatory and inhibitory neurotransmitters in the brain, leading to hyperexcitability and synchronized firing of neurons. Genetic mutations, brain injury, or other factors affect ion channels and neurotransmitter receptors in the brain. These changes can increase the excitability of neurons and make them more likely to fire abnormally (4, 6). Inflammatory changes in the brain, such as the activation of microglia and the release of cytokines, can contribute to seizure development and propagation (7–9). Additionally, reactive oxygen species and other oxidizing agents can damage neurons and increase their excitability, potentially leading to seizures (10, 11).

The potential relationship between canine seizures and cognitive dysfunction syndrome (CDS), a condition characterized by cognitive decline similar to Alzheimer’s disease (AD) in humans, has received extensive focus. Dogs with CDS are more likely to experience seizures than healthy dogs. A dog experiencing a seizure exhibited typical symptoms commonly linked with canine dementia. Additionally, dogs with epilepsy displayed a higher prevalence of canine dementia symptoms compared with the control group (12, 13). Seizures and cognitive impairment are interconnected, and they can interact in a vicious cycle. Specifically, seizures can cause damage to the nervous system, including cell death and alterations in neurotransmitters, thereby affecting cognitive function. In addition, CDS and dementia can increase the incidence of seizures by changing the structure and function of the brain (14–18). Previous research revealed that dogs that experienced seizures had a higher risk of developing cognitive impairment than their counterparts. Furthermore, dogs with cognitive dysfunction were more prone to seizures than dogs without cognitive dysfunction (15). Similarly, people with epilepsy have a higher risk of developing dementia than their counterparts, and the risk of dementia increases with the duration of epilepsy (14, 16, 19). There is a complex relationship among seizures, AD, and amyloid-β (Aβ) (20–22). Recent studies suggested that seizures can contribute to the formation of Aβ plaques in the brain and increase Aβ levels in cerebrospinal fluid (CSF), both of which are hallmarks of AD (21, 23, 24). Prior studies illustrated that Aβ can enhance neuronal excitability and contribute to seizures through a variety of mechanisms, including synaptic dysfunction and neuroinflammation (17, 18). In addition, recent studies identified a potential link between Aβ and epilepsy-associated comorbidities, such as cognitive impairment and depression (18, 25). These studies suggested a bidirectional relationship between seizures and Aβ accumulation in the brain. Specifically, seizures can contribute to the formation of Aβ plaques, and Aβ accumulation can increase the risk of seizures.

Currently, the diagnosis of epilepsy is based on clinical signs, history, and the elimination of other potential causes of seizures (1, 3). Advanced imaging techniques, such as MRI and CT, can provide valuable information about the underlying causes of seizures. Proteomic analysis is a powerful tool that can be used in the diagnosis and therapeutic monitoring of canine epilepsy. In particular, proteomic analysis can be used to identify potential biomarkers of epilepsy in dogs. By comparing the protein profiles of dogs with and without epilepsy, researchers can identify proteins that are differentially expressed in dogs with epilepsy. These proteins can then be validated as potential biomarkers for use in diagnosis and treatment response assessment and as targets for new drugs (26, 27).

The objectives of this investigation were to determine plasma Aβ levels and identify proteins potentially involved in the pathogenesis of refractory canine epilepsy using proteomic analysis. We demonstrated for the first time that increased plasma Aβ_42_ levels were accompanied by alterations in plasma protein expression in dogs with refractory epilepsy. These findings could provide additional insights into the mechanisms underlying epilepsy. In addition, potential plasma biomarkers obtained via proteomic analysis may be utilized for treatment management.

## 2 Materials and methods

### 2.1 Study population

Eight plasma samples were collected from dogs at the Small Animal Teaching Hospital, Faculty of Veterinary Medicine, Mahidol University (Nakhon Pathom, Thailand). The sample size of four animals in each group was determined using the EpiTools epidemiological calculator,^1^ with a significance level of 5% and a confidence level of 95%. The cohort included four dogs diagnosed with refractory idiopathic epilepsy and four healthy dogs. Idiopathic epilepsy was described as a condition in which dogs aged 6 months to 6 years old experienced their first seizure without any other underlying cause for the seizures without significant abnormalities on minimum data base blood tests (according to the IVETF diagnostic Tier I) (28). All idiopathic epilepsy dogs received appropriate dose of phenobarbital and potassium bromide but become refractory to all medication (29). The population characteristics of dogs in this study have been included in the Supplementary material. The sample collection protocol was approved by the Mahidol University Animal Care and Use Committee (AICUC: MUVS-2020-08-37). Blood samples were collected and centrifuged at 3000 rpm for 10 min. The first portion of the plasma sample was stored at −80°C for proteomic analysis, whereas the second portion was stored at −80°C to assess Aβ_42_ levels using enzyme-linked immunosorbent assay (ELISA).

### 2.2 Sodium dodecyl sulfate–polyacrylamide gel electrophoresis (SDS-PAGE)

The concentrations of plasma proteins were measured using Bradford’s assay. Protein samples from each group were pooled. For protein identification using nano liquid chromatography tandem mass spectrometry (nano-LC–MS/MS), 30 μg of pooled protein were loaded onto a 12% polyacrylamide gel. The protein bands on the gel were stained using Coomassie Brilliant Blue R-250 (Bio-Rad, Hercules, CA, USA) and then de-stained using a solution of 30% ethanol in 10% acetic acid. The gel was scanned using a GS-710 scanner (Bio-Rad, Hercules, CA, USA). For further analysis, the protein bands were divided into 11 segments per lane based and cut into pieces. The gel band excision technique was based on the ability to separate different protein sizes physically on the gel and did not cut in the middle of the protein bands. Each individual piece was then subjected to tryptic digestion.

### 2.3 In-gel digestion

Gel pieces were equilibrated using absolute acetonitrile and 50 mM NH_4_HCO_3_. Disulfide bonds were reduced using 4 mM dithiothreitol in 50 mM NH_4_HCO_3_ for 10 min at 60°C and alkylated in 250 mM iodoacetamide in 50 mM NH_4_HCO_3_ for 30 min at room temperature in the dark. The gel pieces were dehydrated twice in absolute acetonitrile for 15 min each and allowed to air-dry. Next, the gel pieces were subjected to trypsin digestion in 50 mM NH_4_HCO_3_ overnight at 37°C. The resulting tryptic peptides were extracted from the gel using absolute acetonitrile. Finally, the peptide mixtures were dried using a speed vacuum and stored at −80°C until analysis by nano-LC–MS/MS.

### 2.4 Analysis of peptide patterns by nano LS-MS/MS

Extracted peptides were dissolved in 0.1% formic acid in LC/MS-grade water. Each sample was injected into the UltiMate 3,000 RSLCnano System (Thermo Fisher Scientific, Waltham, MA, USA). Peptide separation was performed using a C18 column at a flow rate of 300 nL/min. Mobile phase A consisted of 0.1% formic acid in water, whereas mobile phase B consisted of 80% acetonitrile in 0.1% formic acid. The eluent was then infused into a microTOF-Q mass spectrometer (Bruker Daltonics, Billerica, MA, USA). The mass spectra from the mass spectrometry (MS) and tandem MS covered the mass ranges of m/z 400–2000 and m/z 50–1,500, respectively.

### 2.5 Bioinformatics analysis

A mascot generic file (.mgf) was generated using DataAnalysis 3.4 version software. Mascot Daemon version 2.3.2 (Matrix Science, London, UK) was used to identify the proteins. Identification and quantification of the proteins were performed against an NCBInr database (March 02, 2023) specific for dogs. Protein abundance was determined by peptide count analysis using the exponentially modified protein abundance index (emPAI) value (30). The emPAI is a label-free approach for protein quantification. It provides a relationship of direct proportionality between the numbers of observed and expected peptides (30). This technique could inform the relative quantification of each protein in the protein mixture which is more informative than crude protein concentration assay. Three biological replications were performed. Proteins with significantly different expression in the two groups were used to perform clustering analysis. A Venn diagram was used to illustrate differences in protein expression between the groups.

#### 2.5.1 Gene ontology (GO) annotation

Proteins were classified by GO annotation based on three categories: biological processes, cellular components, and molecular function. This analysis helps to understand the biological functions and processes associated with the differentially expressed proteins.

#### 2.5.2 Enrichment of pathway analysis

The protein–protein interactions between differentially expressed proteins in a protein map were assessed using the Search Tool for the Retrieval of Interacting Genes (STRING) database.^2^ This database provides information about known and predicted interactions between proteins, thereby assisting in the exploration of protein networks and potential functional relationships. Next, the Kyoto Encyclopedia of Genes and Genomes (KEGG) database^3^ was used to classify differentially expressed proteins into hierarchical categories. Pathways with false discovery rates smaller than 0.05 were considered statistically significant. Protein function was identified using the UniProt database.^4^

### 2.6 Determination of Aβ_42_ levels by ELISA

To quantify Aβ_42_ levels in plasma, specific sandwich ELISA kits designed for human Aβ_42_ (Elabscience®, Wuhan, China) were used following the manufacturer’s instructions. Several evidences in canine studies have utilized human Aβ_42_ ELISA kit across species (31, 32) due to the identical amino acid sequence of Aβ_42_ between humans and dogs (33). Briefly, ELISA plates were coated with 100 μL of each plasma sample. Then, biotinylated antibody was added to the plates, followed by incubation for 1 h. After several wash steps, HRP conjugate working solution was added to each well. The wash step was repeated, and substrate solution was added to each well. The reaction was stopped by the addition of stop solution. The absorbance of the samples was measured at a wavelength of 450 nm.

### 2.7 Statistical analysis

The statistical significance of differences between the groups was determined using a paired non-parametric Student’s *t*-test. Statistical calculations were performed using GraphPad Prism version 5.0 and significance was indicated by *p* < 0.05.

## 3 Results

### 3.1 Plasma levels of Aβ_42_ in dogs with epilepsy and healthy dogs

**Figure 1:**
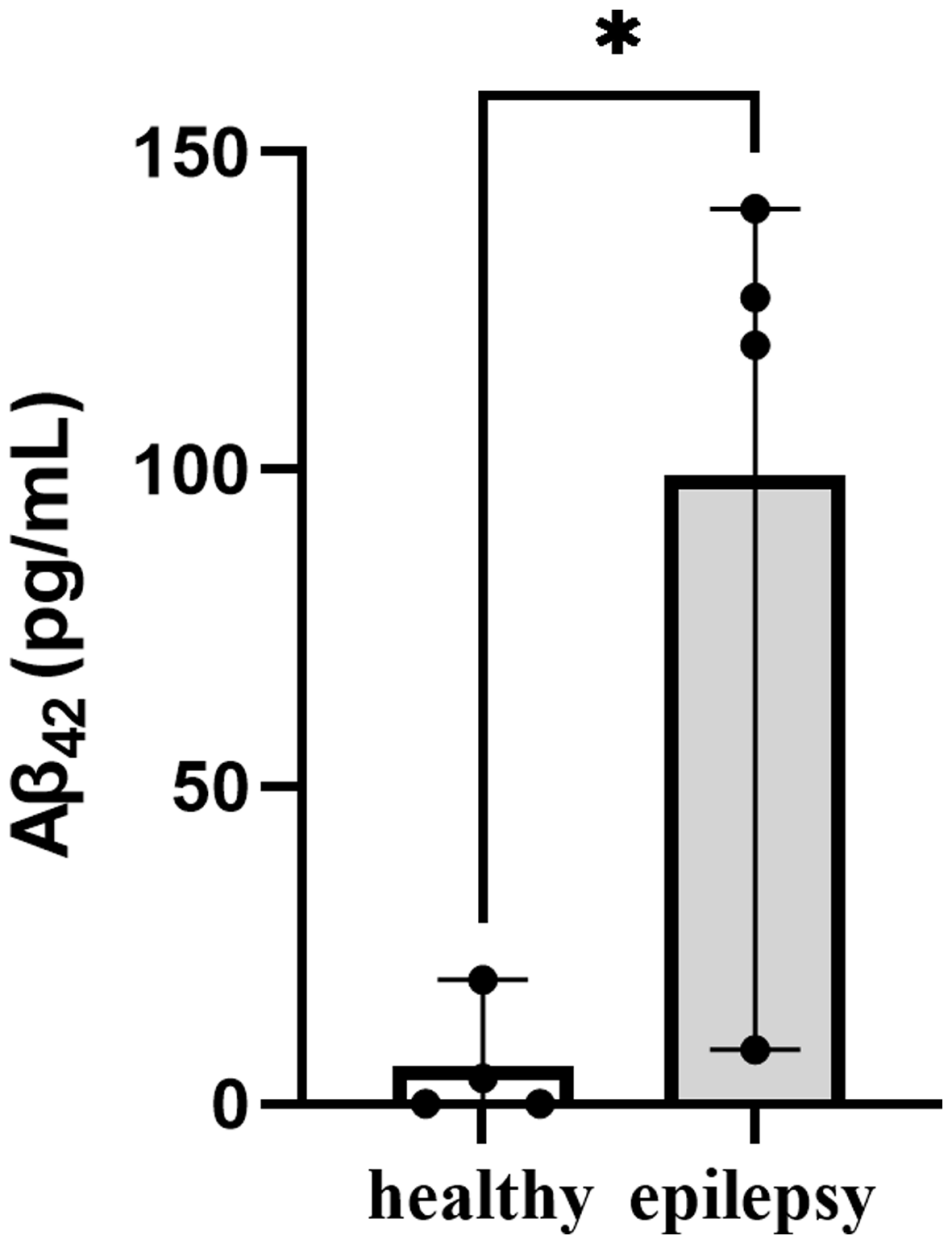
Plasma Aβ_42_ levels in the healthy control and epilepsy groups. *P < 0.05.

The mean plasma Aβ_42_ level with refractory idiopathic epilepsy was significantly greater levels when compared to normal dogs (99 pg/mL vs. 5.9 pg/mL, *p* < 0.05, Figure 1). Similarly, it was observed that dogs with cognitive impairment exhibited increased levels of plasma Aβ_42_ (31, 32, 34).

### 3.2 Plasma proteomics

**Figure 2:**
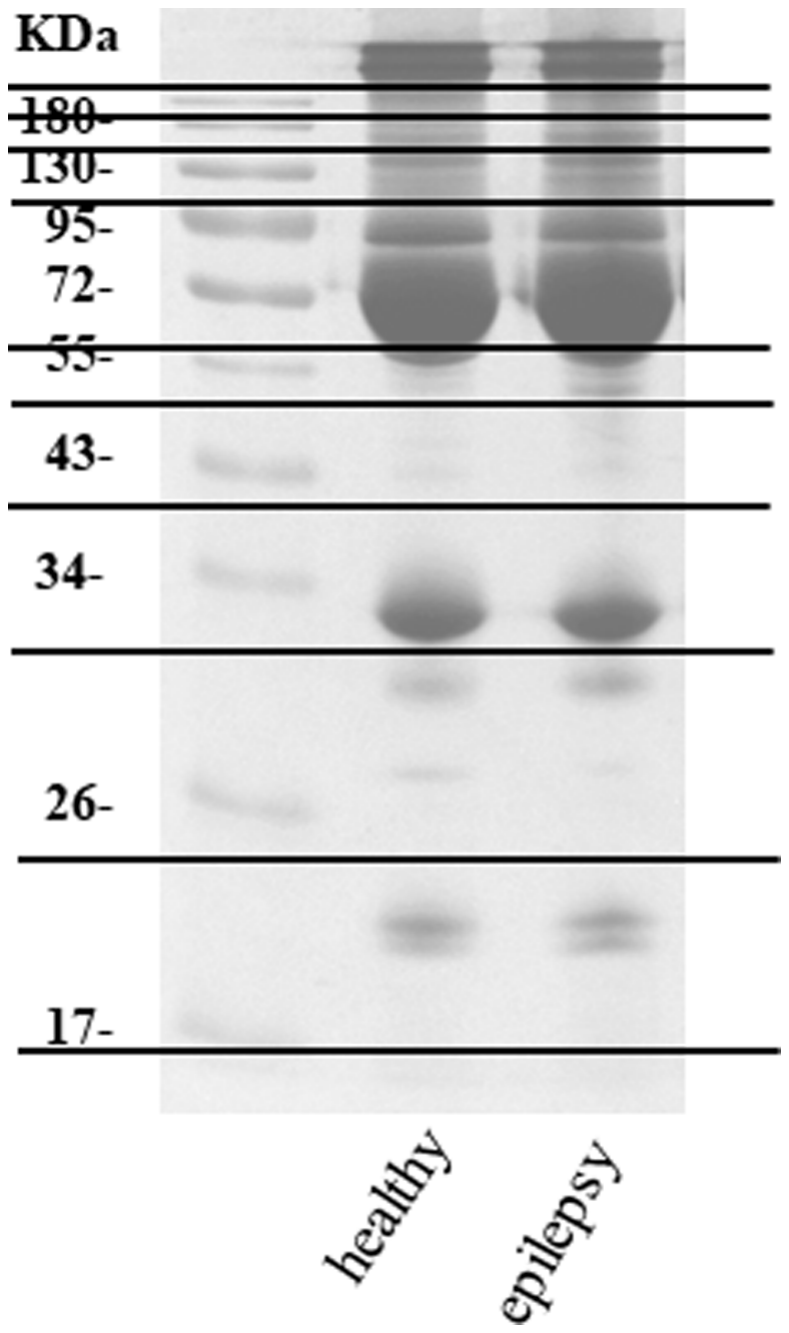
Protein separation one-dimensional gel electrophoresis.

**Figure 3:**
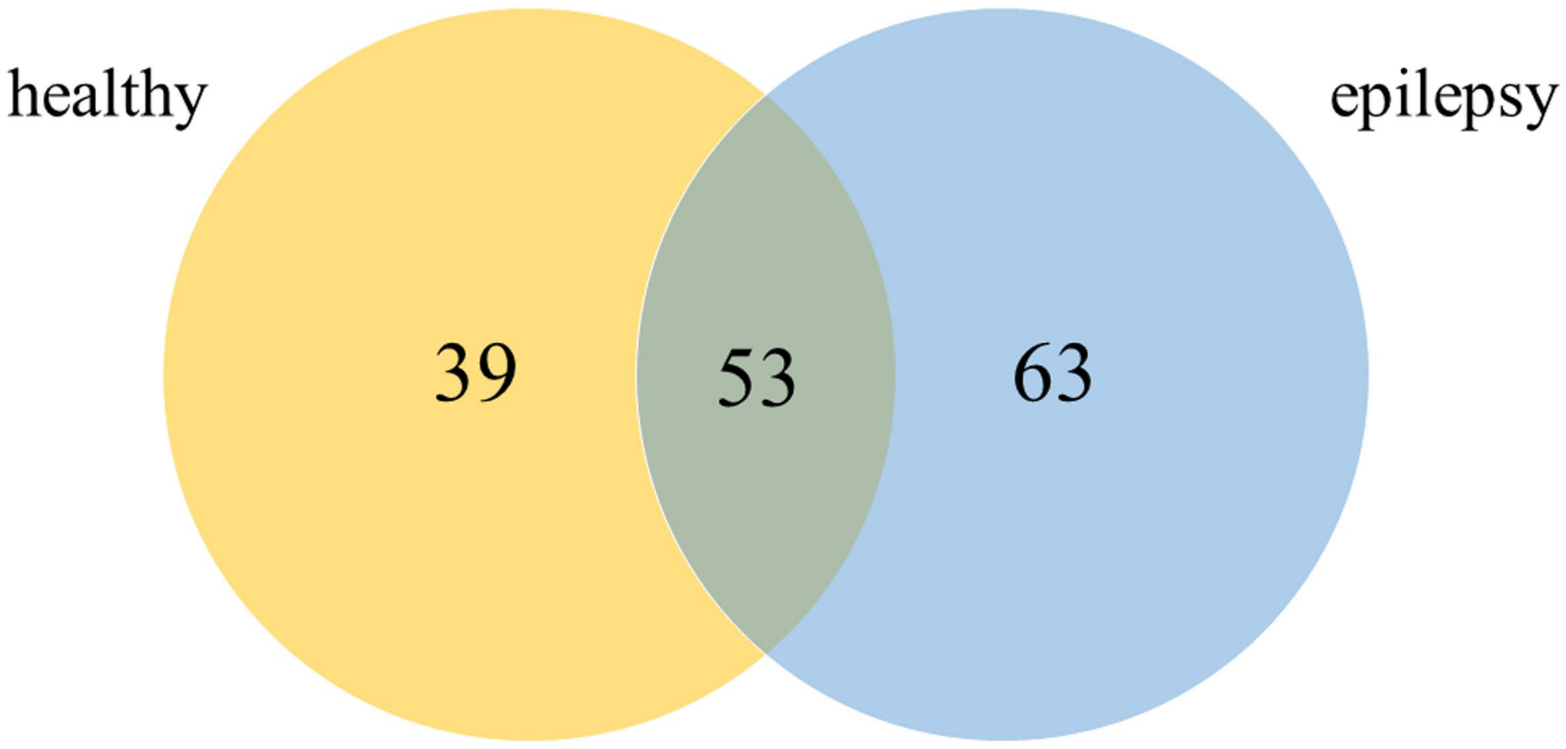
Venn diagram of detected proteins in the healthy control and epilepsy groups (*Canis* spp.).

Plasma from dogs in the healthy control and epilepsy groups were pooled and prepared to measure differential protein expression using a proteomic approach. The protein bands in one-dimensional gel electrophoresis of both groups were separated into 11 pieces prior to in-gel trypsin digestion (Figure 2). Of the 155 canine proteins examined, 53 (34.19%) were commonly expressed in dogs in both groups (Figure 3).

### 3.3 Identification of differentially expressed proteins in plasma

The downregulated and upregulated proteins in dogs with epilepsy compared to those in healthy dogs were specifically involved in several biological processes. Differential protein expression was demonstrated via semi-quantification by selecting the altered proteins with at least two replicates (35, 36). Of the 155 examined proteins, 40 displayed at least 1.5-fold differences in expression between the healthy control and epilepsy groups according to the emPAI values.

### 3.4 GO and pathway enrichment analyses of proteins

**Figure 4:**
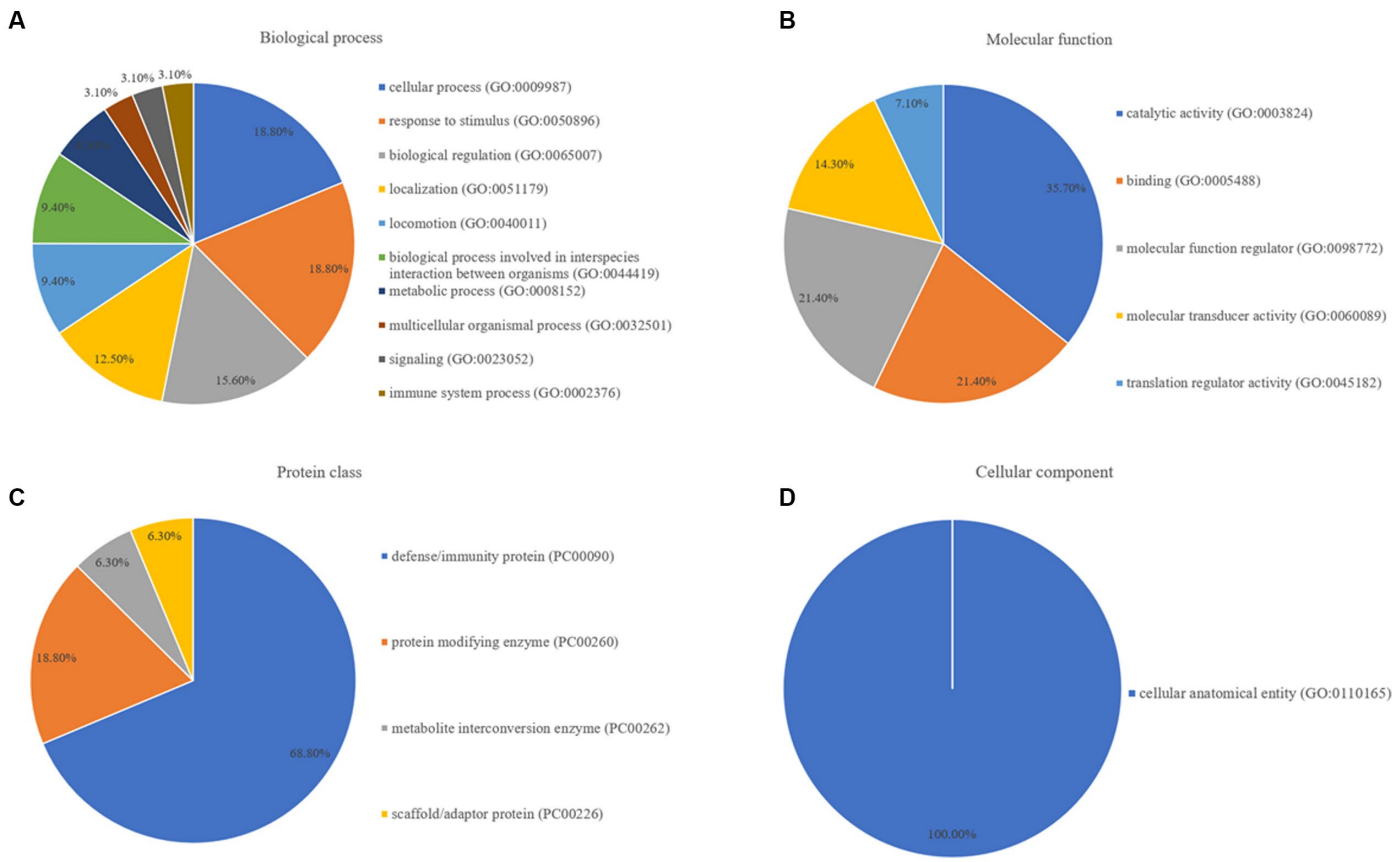
GO annotation of; **(A)** biological process, **(B)** molecular function, **(C)** protein class, and **(D)** cellular component, for differentially expressed proteins between the healthy control and epilepsy groups.

Pathway enrichment analysis was performed to map the proteins onto GO databases via PANTHER using three primary categories: biological process, protein class, and molecular function. In the GO molecular function category, the differentially expressed proteins between the healthy control and epilepsy groups were divided into four groups: biological process, molecular function regulator, protein class, and cellular component (Figure 4). Among the differentially expressed proteins in the epilepsy group, six, six, and five were mapped within cellular processes, response to stimuli, and biological regulation, respectively, in the biological process category (Figure 4A). Five proteins were clustered within catalytic activity and five proteins were involved in binding, molecular function regulation, and molecular transducer activity in the molecular function category (Figure 4B). Eleven proteins participated in defense/immunity protein in the protein class category (Figure 4C), and eight proteins participated in cellular anatomical entity in the cellular component category (Figure 4D).

These results illustrated compared to the findings in healthy dogs, biological processes such as cellular activities, localization, response to stimuli, and critical molecular activities such as catalytic activity, molecular function regulation, and binding were disrupted in dogs with epilepsy. Defense/immunity proteins were the most dramatically altered proteins throughout the development of epilepsy.

### 3.5 Proteins of interest and subcellular distribution of differentially expressed proteins

Twelve proteins were upregulated in the epilepsy group, whereas 10 proteins were downregulated (Table 1). To predict the cellular functions of differentially expressed proteins, their localization was analyzed in this study. Using subcellular location analysis with data from the UniProt annotation database, the distribution of the proteins was as follows: secreted, 14 (63.64); endoplasmic reticulum, 1 (4.55%); cell membrane, 3 (13.64%); cytoplasm, 4 (18.18%); mitochondria, 1 (4.55%); cytoskeleton, 2 (9.09%); and extracellular space, 1 (4.55%, Table 1). The proteins of interest in dogs, identified from the MS/MS data reported here, exhibited a confidence level above 95%, as determined by the false discovery rate.

**Table 1:** Differentially expression proteins in dogs with epilepsy and healthy controls.

Upregulated proteins in the epilepsy group					
Protein accession number	Protein name	Subcellular distribution	Protein function	Fold change	*p*
A0A8C0L8V9	Haptoglobin	Extracellular space, secreted	Antioxidant, acute phase response	6.94	0.257
A0A8C0KKB1	α-1-antitrypsin-like	Endoplasmic reticulum	Irreversibly inhibits trypsin, chymotrypsin and plasminogen activator	2.22	0.005
A0A8C0QTZ3	Fibrinogen gamma chain	Secreted	Functions in hemostasis	2.19	0.071
A0A8C0QD48	Immunoglobulin heavy constant mu	Cell membrane, secreted	Primary defense mechanisms	2.00	0.381
A0A8C0N3V5	Gelsolin	Cytoplasm, cytoskeleton, secreted	Calcium-regulated, actin-modulating protein	Only expressed in the epilepsy group	0.096
A0A8C0KQR0	Pentatricopeptide repeat domain 3	Mitochondria	Mitochondrial RNA-binding protein that has a role in mitochondrial translation	Only expressed in the epilepsy group	0.092
A0A1K0GGH0	Globin A2	N/A	Involved in oxygen transport	Only expressed in the epilepsy group	0.095
A0A8C0RS56	Complement factor B	Secreted	Alternate pathway of the complement system	Only expressed in the epilepsy group	0.116
A0A8C0M9Q8	Ig α-1 chain C region	Secreted, cell membrane	Defends against infection and prevents the access of foreign antigens	Only expressed in the epilepsy group	0.019
Q7M321	Plasmin (fragment)	Secreted	Dissolves the fibrin of blood clots and acts as a proteolytic factor	Only expressed in the epilepsy group	0.147
A0A8C0JUJ8	Complement factor H	Secreted	Complement activation modulator, soluble inhibitor of complement	Only expressed in the epilepsy group	0.344
A0A8C0L3D1	Ceruloplasmin	Secreted	Ferroxidase activity oxidizing Fe^2+^ to Fe^3+^	Only expressed in the epilepsy group	0.102
Downregulated proteins in the epilepsy group					
A0A8C0K0Q8	α2-macroglobulin	Secreted	Inhibits all four classes of proteinases	9.50	0.134
A0A8C0NNW8	GLOBIN domain-containing protein	N/A	Involved in oxygen transport	1.98	0.018
A0A8C0JZK1	Pregnancy zone protein-like	Secreted	Inhibits all four classes of proteinases	1.88	0.128
A0A8C0NL73	Hemoglobin subunit beta	Secreted	Involved in oxygen transport	Only expressed in the healthy control group	0.188
A0A8C0LTI4	Vitamin D binding protein	Secreted	Involved in vitamin D transport and storage, scavenging of extracellular G-actin	Only expressed in the healthy control group	0.355
A0A8I3PH35	GLOBIN domain-containing protein	N/A	Involved in oxygen transport	Only expressed in the healthy control group	0.002
A0A8P0NMZ6	Keratin, type II cytoskeletal 1	Cell membrane, cytoplasm	Regulate the activity of kinases	Only expressed in the healthy control group	0.259
A0A8C0L7B0	GCN1 activator of EIF2AK4	Cytoplasm	Acts as a positive activator of the GCN2 protein kinase activity	Only expressed in the healthy control group	0.259
A0A8C0M1F4	α-1-B glycoprotein	N/A	Related to inflammation, including binding of pathogens and modulating white blood cells activity	Only expressed in the healthy control group	0.092
A0A8C0K5X2	Gelsolin	Cytoplasm, cytoskeleton, secreted	Calcium-regulated, actin-modulating protein	Only expressed in the healthy control group	0.113

### 3.6 KEGG function analysis of differentially expressed proteins

**Figure 5:**
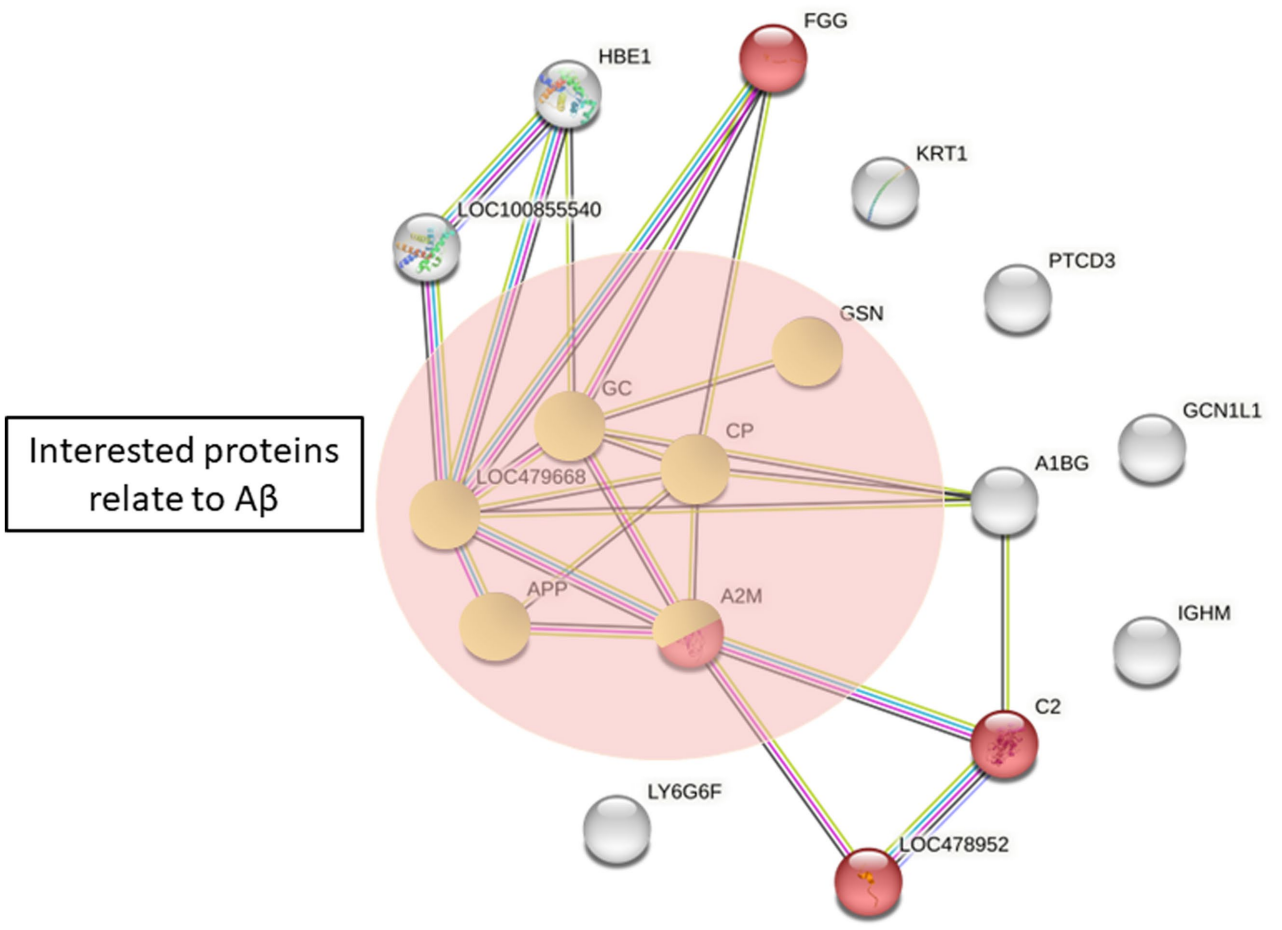
Protein differences between the healthy control and epilepsy group by STRING. Red = complement and coagulation cascades (KEGG pathway). Proteins in green circles are proteins of interest related to Aβ_42_ (APP, amyloid beta A4 protein; A2M, α2-macroglobulin; CP, ceruloplasmin; GSN, gelsolin; GC, vitamin D binding protein; LOC479668, haptoglobin).

To explore the potential proteins involved in the pathogenesis of canine epilepsy and Aβ_42_ metabolism, we performed pathway analysis using STRING version 11.0. Total protein changes in the epilepsy group in comparison with the healthy control group were expanded to illustrate evidence of interactions, resulted in the identification of 17 proteins. We compared this protein set to those in the GO and KEGG databases. The proteins related with Aβ were haptoglobin (HP), α2-macroglobulin, ceruloplasmin, complement factor H (CFH), and gelsolin. The proteins potentially associated with the pathogenesis of epilepsy are involved in complement and coagulation cascades (Figure 5).

## 4 Discussion

A growing body of research has suggested an association between AD and epilepsy (18). Canine refractory epilepsy have a higher risk of developing CDS at a younger age than normal dogs (12). Multiple studies in animals and humans have revealed an association between Aβ and epilepsy (37–39). In our study, we first demonstrated significantly higher plasma Aβ levels in dogs with refractory epilepsy. Plasma Aβ_42_ levels in healthy dogs in this study were comparable to those recorded in a previous study (10.99 ± 5.45 pg/mL) (31), and plasma Aβ_42_ levels were elevated in age-matched dogs with epilepsy. The plasma proteomic pattern was assessed, and five major proteins potentially involved in the pathogenesis of epilepsy and elevated Aβ levels were identified: haptoglobin (HP), α2-macroglobulin, ceruloplasmin, complement factor H (CFH), and gelsolin.

The interplay between epilepsy and cognitive dysfunction has been studied in dogs. However, the evidence demonstrating that Aβ levels are higher in dogs with than in healthy controls is limited. A previous study found that dogs with severe early-onset epilepsy had higher Aβ accumulation and greater neuronal degeneration in the brain than healthy controls (40). Additionally, substantial evidence has revealed an association between Aβ and epilepsy in animals and humans. For example, the presence of Aβ plaques was associated with an increased frequency and duration of epileptic spiking in APP/PS1 mice (37). Aβ protein levels and their relationship with cognitive function have been investigated in patients with refractory epilepsy by analyzing cortical biopsies from the temporal lobes. The results revealed a strong compelling of Aβ deposits in the biopsied tissues, supporting the existence of Aβ deposits in patients with refractory epilepsy (39). In patients with refractory seizures, increased Aβ precursor protein was detected in temporal lobe or hippocampal sections (41). A study on rats illustrated that epilepsy can lead to increased Aβ expression (42, 43). Seizures contribute to neurodegenerative processes by triggering electrical currents and promoting the production and release of Aβ (37, 44). The presence of Aβ_42_ leads to an increase in neuronal excitability in AD, which subsequently initiates the development of progressive epilepsy (17). Our findings demonstrate that increased levels of Aβ_42_ in dogs with treatment-resistant seizures may be attributed to prolonged epileptiform discharges in the brain, leading to the development of cognitive impairment. Further research is needed to fully understand the mechanisms underlying this relationship and to identify potential therapeutic targets.

Plasma HP, which is primarily produced by hepatocytes in the liver, is a particularly important hemoglobin-binding protein that removes hemoglobin from the circulation (45). Various additional functions of HP have been discovered, including serving as an acute phase 2-acid response glycoprotein, an antioxidant of apolipoprotein E (APOE), an anti-inflammatory protein, and an Aβ clearance facilitator (46, 47). In the current study, HP was significantly upregulated in the epilepsy group compared to that in the healthy control group. A potential association of HP with neurological disorders was discovered. Significant upregulation of HP, interferon gamma, and interleukin-1β was associated with refractory epilepsy, as determined by proteomic analysis of plasma isolated from children with refractory epilepsy (48). Serum HP levels were significantly higher in patients with idiopathic seizures than in healthy controls (49). Similarly, proteomic analysis of CSF from dogs with recurrent epileptic seizures revealed a significant increase in HP levels, suggesting that HP participates in disruption of the blood–brain barrier, which is potentially linked to the inflammatory response triggered by seizures within the brain (26). Patients with AD have significantly higher serum HP levels than healthy controls. Additionally, a significant positive correlation between the serum HP level and the severity of cognitive impairment was observed in patients with AD (47, 50). This association is believed to occur through the modification of the effects of APOE, another genetic factor implicated in AD progression (46). Although research on the relationship among HP, epilepsy, and Aβ is currently limited, the present findings suggest possible links among these factors.

In the present study, ceruloplasmin was only detected in dogs with epilepsy. Ceruloplasmin is a circulating copper-binding protein that participates in copper homeostasis, oxidative stress, and neuroinflammation, and its expression is increased during the acute phase response (51, 52). Evidence suggests that plasma ceruloplasmin levels reflect its levels in the brain (53). There is some evidence that ceruloplasmin is involved in seizure activity, although the exact relationship is not fully understood (28, 54, 55). Plasma ceruloplasmin concentrations and oxidase activity were substantially higher in adults with epilepsy than in age- and gender-matched controls (28). Ceruloplasmin mRNA expression in the peripheral blood was significantly higher in patients with refractory epilepsy than in drug-responsive patients and healthy controls. The researchers additionally found a link between increased ceruloplasmin expression and different treatment strategies, potentially revealing a resistance mechanism for combination medications used to treat refractory epilepsy (28). The relationship between epilepsy and partial duplication of the ceruloplasmin gene in mice in epilepsy, which is coinherited with seizures, was studied, and duplication of the gene was associated with increased ceruloplasmin mRNA expression and ceruloplasmin oxidase activity (55). In addition, the role of ceruloplasmin in Aβ metabolism has been studies. There is evidence that High CSF ceruloplasmin levels in patients with AD and underlying Aβ pathogenesis was related to faster cognitive deterioration (51). These studies suggest the involvement of ceruloplasmin in seizure activity and Aβ metabolism through its roles in oxidative stress and copper homeostasis.

In the present study, CFH was only detected in the plasma of dogs with epilepsy. The protein regulates complement system activity and modulates inflammation in the brain and periphery. Despite being mainly synthesized and secreted by the liver, CFH has been found in different tissues, including the brain (56). Recent studies have suggested a possible link between mutations or dysregulation of CFH and epilepsy. Ten complement analytes were measured in patients with focal or generalized epilepsy, and plasma CFH levels were significantly higher in such patients than in controls (56). Although the exact mechanisms underlying the relationship between CFH and seizures are not fully understood, it is believed that dysregulation of the complement system contributes to neuroinflammation and neuronal damage, leading to an increased risk of seizures. In addition, recent studies have attempted to demonstrate the possible relationship between CFH and Aβ in AD (57, 58). Plasma CFH levels were reduced in patients with late-onset AD, and this reduction was associated with serum C-reactive protein levels. Unfortunately, it was recently suggested that plasma CFH is not an appropriate biomarker for AD (57).

α2-Macroglobulin is a broad-spectrum proteinase inhibitor and an acute phase protein of the innate immune system that is extensively found in the plasma of animals and distributed in various body fluids, including CSF (59–61). The plasma levels of α2-macroglobulin and cytokines are elevated in patients with neurodegenerative diseases including AD and Parkinson’s disease (61). It has been proposed that increased activity of α2-macroglobulin has a role in AD pathogenesis involving Aβ plaque accumulation (62). Plasma α2-Macroglobulin functions as a carrier protein and specifically binds to soluble Aβ and facilitates its degradation (63–65). This process may help in the clearance of Aβ from tissues, including the brain. The current study showed a reduced expression of α2-macroglobulin in seizure group, which may result in a diminished clearance of Aβ. This can explain for why elevated plasma levels of Aβ were identified in the seizure group.

In the present study, plasma gelsolin was only detected in healthy dogs. Gelsolin is a calcium and phosphatidylinositol 4,5-bisphosphate–regulated protein with actin-binding properties that is involved in various cellular processes, including cell signaling, inflammation, and cytoskeletal organization (66). There is evidence of the potential of gelsolin as a biomarker for epilepsy, with people with epilepsy having considerably lower CSF levels of gelsolin than healthy controls. Gelsolin protein levels were similarly reduced in the temporal lobe in patients with epilepsy (67). Seizure-induced damage to hippocampal pyramidal neurons was exacerbated in gelsolin-deficient adult mice, suggesting that gelsolin activates N-methyl-D-aspartate receptors and voltage-dependent calcium channels, leading to pathophysiological events (68). In addition, gelsolin m expression was lower in the hippocampus of mice with epilepsy and seizures than in mice without seizures (69). Gelsolin is also considered to be involved in AD and the regulation of Aβ levels according to the finding that diverse gelsolin alterations are connected with the progression of AD (70, 71). Gelsolin administration or overexpression induced a considerable reduction in the amyloid burden and Aβ level in AD transgenic mice (70, 72). Greater plasma and CSF gelsolin concentrations were detected in patients with AD than in controls, and a positive association was found between gelsolin and Aβ_42_ levels in CSF. The increase in plasma gelsolin levels is most likely a compensatory reaction in AD (71).

**Figure 6:**
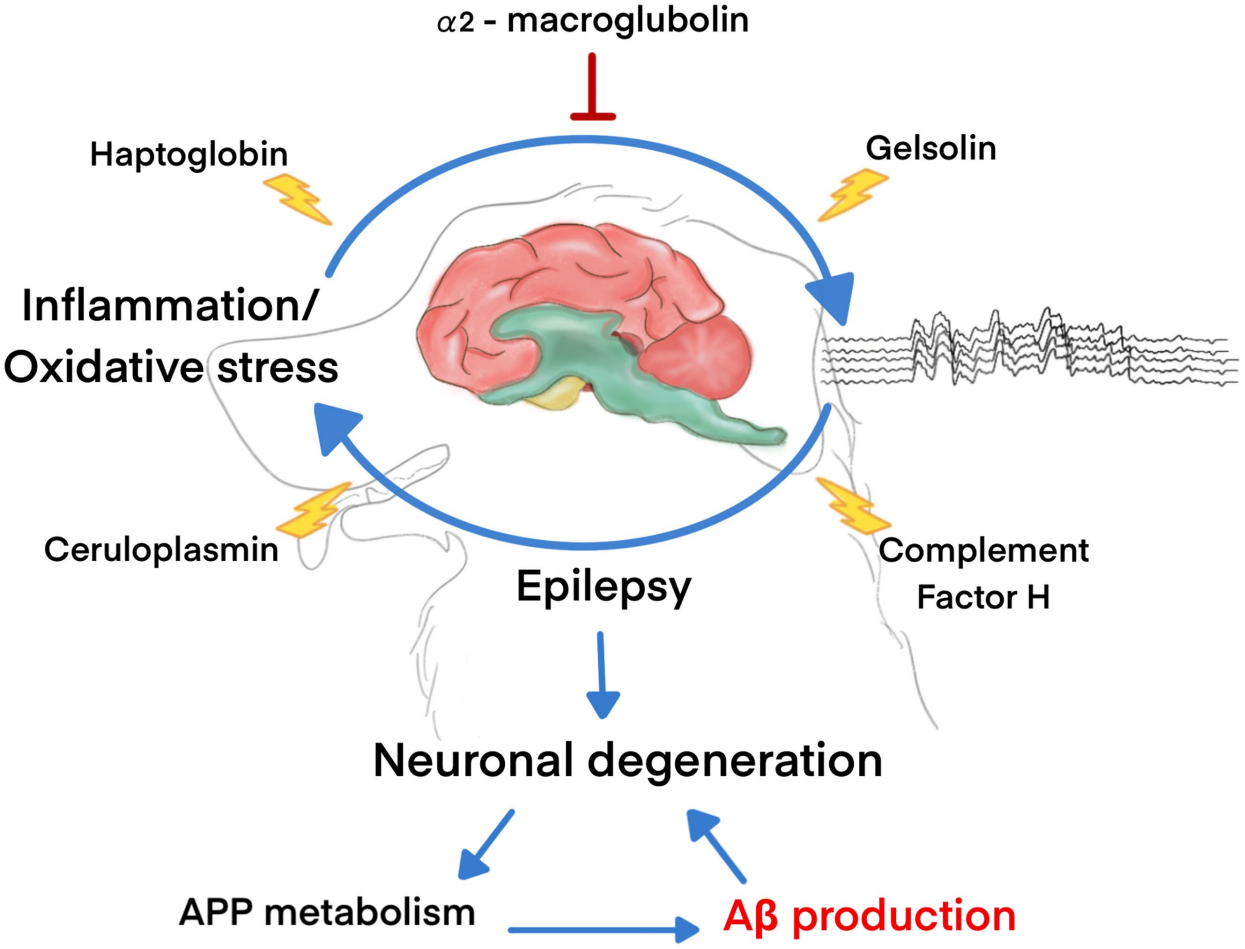
The molecules associated with Aβ production in dogs with epilepsy.

In conclusion, we first demonstrated that plasma Aβ_42_ levels were significantly higher in dogs with refractory epilepsy than in healthy dogs. The plasma proteomic pattern was identified, and five major proteins potentially involved in the pathogenesis of epilepsy and control of Aβ levels, including HP, α2-macroglobulin, ceruloplasmin, CFH, and gelsolin, were identified. HP and α2-macroglobulin are proteins involved in acute phase, immunological, and inflammatory responses. HP expression was substantially elevated in the epilepsy group, indicating relationships with early neuronal injury and enhanced Aβ clearance. Conversely, α2-macroglobulin was significantly upregulated in the healthy control group, and the role of α2-macroglobulin in seizure etiology remains unknown. CFH and ceruloplasmin were only detected in the plasma of dogs with epilepsy, suggesting potential roles in neuroinflammation and seizures. Contrarily, gelsolin, which is involved in cellular processes and cytoskeletal organization, was only found in the plasma of healthy dogs. However, the exact mechanisms underlying these relationships and their implications in canine epilepsy require further research (Figure 6). Although the sample size of animals in the study is sufficient to examine the difference in plasma levels of β-amyloid_42_ between healthy dogs and those with refractory epilepsy, the proteomics analysis is limited by the small number of dogs. The present study demonstrated that proteomic analysis has the potential to identify novel biomarkers, mechanisms, and therapeutic targets for seizure disorders. However, further research is needed to validate these biomarkers and overcome the challenges associated with proteomic analysis in neurological disorders.

## Additional information

The study was approved by the Committee on the Care and Use of Laboratory Animals in the Faculty of Veterinary Science, Mahidol University, Thailand (approval number: MUVS-2020-08-37). The owners were informed and signed in the consent form.

## Data availability statement

The datasets presented in this study can be found in online repositories. The names of the repository and accession number can be found here: https://www.ebi.ac.uk/pride/, PXD043962.

## Ethics statement

The animal studies were approved by Committee on the Care and Use of Laboratory Animals in the Faculty of Veterinary Science, Mahidol University, Thailand (approval number: MUVS-2020-08-37). The studies were conducted in accordance with the local legislation and institutional requirements. Written informed consent was obtained from the owners for the participation of their animals in this study.

## Author contributions

SP: Conceptualization, Data curation, Investigation, Validation, Visualization, Writing – original draft, Writing – review & editing. BC: Conceptualization, Methodology, Resources, Supervision, Validation, Visualization, Writing – original draft, Writing – review & editing. OR: Data curation, Resources, Writing – review & editing. DC: Conceptualization, Funding acquisition, Investigation, Methodology, Supervision, Validation, Visualization, Writing – original draft, Writing – review & editing.

## Footnotes

^1^ http://epitools.ausvet.com.au/content.php?page=home

^2^ https://string-db.org

^3^ https://www.genome.jp

^4^ www.uniprot.org
