# Supplementary material for: Protein profiling and assessment of amyloid beta levels in plasma in canine refractory epilepsy
Source: Front Vet Sci. 2023 Dec 21;10:1258244. doi: 10.3389/fvets.2023.1258244 (PMC10772147; doi:10.3389/fvets.2023.1258244)
Supplement: Supplementary file 1 [file Table_1.DOCX]

**Table 1** The population characteristics of dogs in this study

| **Group** | **Number** | **Breed** | **Sex** | **Ages (months)** | **Anti-epileptic drugs** |
| --- | --- | --- | --- | --- | --- |
| IE | 1 | Labrador Retriever | Male | 66 | Phenobarbital  Potassium bromide  Gabapentin |
|  | 2 | Beagle | Female | 18 | Phenobarbital  Potassium bromide  Levetiracetam |
|  | 3 | Jack Russell Terrier | Male | 39 | Phenobarbital  Potassium bromide  Levetiracetam |
|  | 4 | Jack Russell Terrier | Male | 39 | Phenobarbital  Potassium bromide  Levetiracetam |
| Adult | 5 | Mongrel | Male | 36 | None |
|  | 6 | Mongrel | Female | 14 | None |
|  | 7 | Mongrel | Male | 40 | None |
|  | 8 | Mongrel | Male | 12 | None |

IE = Idiopathic epilepsy
